# Laparoscopic Dismembered Repair in Two Patients with Retrocaval Ureter

**DOI:** 10.1055/s-0040-1705156

**Published:** 2020-04-23

**Authors:** Yuri Cavaleri, Giuseppe Farullo, Simona Gerocarni Nappo, Paolo Caione

**Affiliations:** 1 Department of Surgery, Urology UOSD, University of Rome Tor Vergata, Roma, Lazio, Italy; 2 Department of Nephrology and Urology, Bambino Gesù Children's Hospital, IRCCS, Rome, Italy; 3 Division of Pediatric Urology, Bambino Gesù Children's Hospital, IRCCS, Rome, Italy

**Keywords:** retrocaval ureter, ureteroureterostomy, laparoscopy

## Abstract

Retrocaval ureter (RCU) or circumcaval ureter is a rare cause of congenital hydronephrosis. The surgical correction of RCU should be performed in all patients with obstruction and hydronephrosis symptoms, lumbar pain, urinary tract infections, hematuria, or urolithiasis. Traditionally, an open surgical approach was used for the treatment of RCU. Nowadays, surgical correction of these anomalies is performed using minimally invasive techniques. We report on two cases treated with our standardized laparoscopic technique using only three 5-mm trocars. The proposed approach could be considered as the first-line treatment for RCU.

## Introduction

Retrocaval ureter (RCU) or circumcaval ureter is a rare cause of congenital hydronephrosis. Although its exact incidence remains unknown, its estimated incidence is 1 in 1,100 live births per year, 1 with a male-to-female ratio of 3:1. 2 RCU occurs due to an unusual persistence of the right subcardinal vein positioned ventrally to the ureter and developed in the definitive inferior vena cava (IVC). However, it is sometimes associated with other IVC abnormalities. 3 The lumbar ureter compression can indicate several degrees of obstruction without specific clinical presentation; therefore, many patients may be completely asymptomatic. Its clinical manifestation is usually recognized from the second to the fifth decade of life, with flank pain and recurrent urinary tract infections as the most common symptoms and hematuria and urolithiasis as the less common symptoms. RCU may also result in long-term renal function impairment. 4 Ultrasonography, multislice computed tomography, contrast-enhanced magnetic resonance imaging (MRI), and mercapto-acetyl-triglycine (MAG3) scintigraphy are the main diagnostic tools. For many years, the gold standard treatment for RCU has been ureteral transposition with uretero-ureteric anastomosis by open surgery. However, in the last two decades, laparoscopic ureteroureterostomy has been proposed as a feasible option. 1 5 Here, we performed a minimally invasive laparoscopic approach in two patients by adopting a standardized technique and demonstrated its safety and efficacy.

## Case Report

**Fig. 1:**
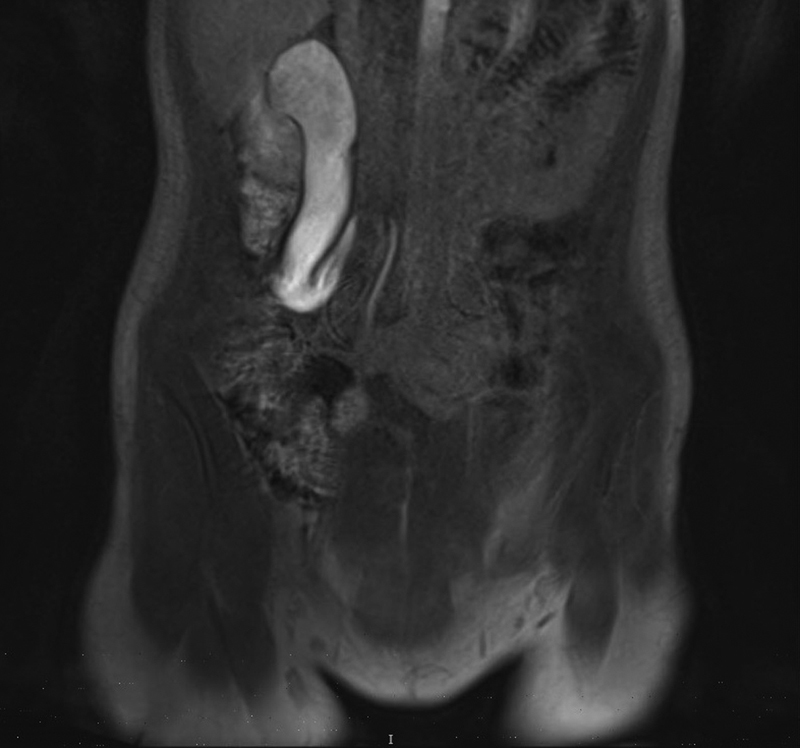
Coronal view of the magnetic resonance image of the typical type 1 retrocaval ureter variant with S-shaped deformity of right lumbar ureter with hydronephrosis and dilatation of the associated proximal ureter (case 2).

**Fig. 2:**
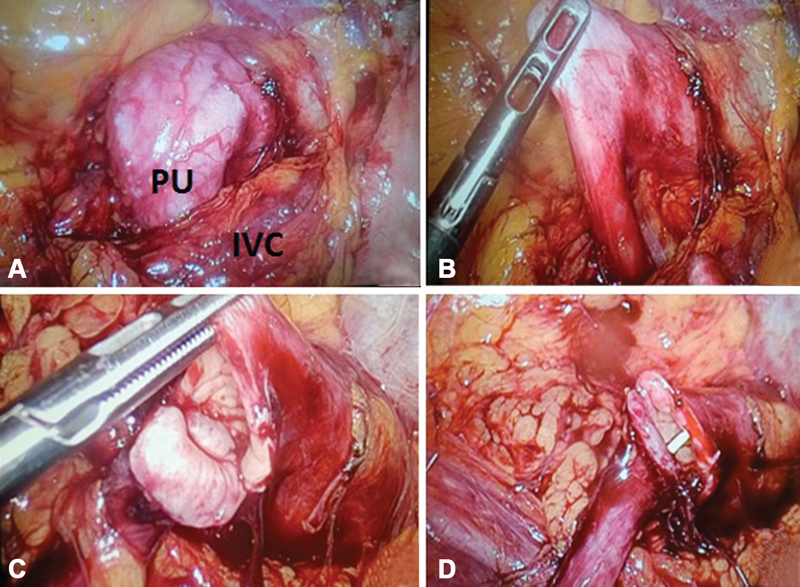
Intraoperative images of the proximal dilated right ureter (case 2). ( **A** ) Dilated proximal ureter (PU) crossing the inferior vena cava (IVC). ( **B** ) Mobilization of the PU with narrowed segment under the IVC. ( **C** ) Stenotic tract of the ureter excised. ( **D** ) Uretero-ureteric anastomosis after spatulation.

From January 2016 to January 2018, two female patients with symptomatic RCU were examined. Their ages at the time of surgery were 22 and 18 years. Body weights were respectively 72 and 56 kg. Patient 1 underwent augmentation enterocystoplasty with bilateral ureteral reimplantation for spinal dysraphism at the age of 5 years. The RCU was not recognized at that time, because the refluxing ureter was grossly dilated and tortuous. Recently, she presented recurrent episodes of right flank pain and two episodes of acute pyelonephritis, which were treated with antibiotics. No reflux was present in the cystogram. Patient 2 showed intermittent gross hematuria with several episodes of flank pain since the age of 12 years. Abdominal MRI with three-dimensional reconstruction revealed that both the patients had a typical S-shaped deformity of the right lumbar ureter, according with type 1 variant of RCU 6 ( Fig. 1 ). The renogram for MAG3 renal scan revealed that the affected kidneys of both the patients had good renal function (split renal function of 47 and 45%, respectively) with partially obstructed urine drainage. Patients underwent minimally invasive laparoscopic ureterocaval transposition with dismembered ureteroureterostomy through a transperitoneal approach using three 5-mm trocars. Patients were positioned in 45 degrees left flank position. The pneumoperitoneum was established at 12 mm Hg. The dilated pelvis and right ureter were identified after mobilizing the right colon and opening the Gerota fascia ( Fig. 2A ). The proximal ureter was lifted on a vessel loop, and the distal ureter segment was poorly mobilized in the inter-aorto-caval region ( Fig. 2B ). The ureter was transected proximally to the IVC, and then the stenotic tract was excised ( Fig. 2C ). The uretero-ureteric anastomosis was created using two running sutures (5/0 polyglactin) after adequately spatulating the distal segment. A 4.7-Fr double-J stent was inserted laparoscopically with an antegrade technique through a percutaneous 18-G needle ( Fig. 2D ). A 10-Fr continuous suction drain was placed postoperatively. All procedures were laparoscopically completed within 195 and 172 minutes in patients 1 and 2, respectively, without perioperative complications. In addition, intracorporeal suturing took 79 and 73 minutes, and blood loss was ∼20 and 30 mL in patients 1 and 2, respectively. The postoperative period was uneventful for both patients. The suction drain was removed after 48 and 60 hours from patients 1 and 2, respectively. Subsequently, patients 1 and 2 were discharged on the fourth and fifth postoperative day, respectively. The double-J stent was removed after 4 weeks. Renal ultrasonography and scintigraphy were performed 1 month after the double-J stent removal. Renal ultrasonography showed a resolved pyelocalyceal dilatation at 3-, 6-, and 12-month follow-up. Renal function was stable, and MAG3 renal scan revealed significant increase in urine drainage in both the patients at the 18-month follow-up. Patients remained symptom-free 24 months postoperatively.

## Discussion

RCU is a rare congenital anomaly generally associated with upper urinary tract obstruction and hydronephrosis. Its typical radiological presentation is an S- or sickle-shaped appearance, due to the passage of the ureter at the posterior side of the IVC. 5 According to the radiologic appearance, RCU is classified into two types. 6 In type 1 (low loop, S-shaped), the dilated upper ureteral tract descends from the renal pelvis and then curves upward and medially forming a reversed J appearance in the intravenous pyelogram. The retrograde pyelogram shows a typical S-shaped outline ( Fig. 1 ). The level of ureteral retrocaval segment is most frequently found at the third lumbar vertebrae. In type 2 variant (high loop, sickle-shaped), the renal pelvis and upper ureter lie almost horizontal; therefore, the ureteral retrocaval segment is on the same level. Type 1 variant is more common and usually causes moderate to severe hydronephrosis, whereas type 2 is rarer with less severe hydronephrosis and does not generally cause obstruction. 7 8 Both our cases were recognized as type 1 variant of RCU. The surgical correction of RCU should be performed in all patients with obstruction and hydronephrosis symptoms, lumbar pain, urinary tract infections, hematuria, or urolithiasis. Asymptomatic patients with functionally unobstructed RCUs who may even show hydronephrosis on radiological imaging do not necessarily need immediate surgical treatment. 9 Our two patients presented symptoms of obstruction and hydronephrosis with urinary tract infections. Traditionally, an open surgical approach was used for the treatment of RCU. However, there was wide incision, quite long convalescence period, and delay in return to daily activities. Therefore, surgical correction of these anomalies has been attempted using minimally invasive techniques. In 1994, Baba et al described the first laparoscopic transperitoneal procedure for treatment of RCU. 10 Nowadays, RCU can be approached laparoscopically by performing either trans- or retroperitoneal procedures. A few studies have reported the utility of the laparoscopic transperitoneal approach for the treatment of RCU with consistent positive results. 11 12 13 14 15 According to Ramalingam and Selvarajan, 11 transperitoneal approach is a less time-consuming, relatively easier procedure than the retroperitoneal approach because of larger operation field and very good exposure.

Our technique of RCU management utilizes only three 5-mm trocars, without any preoperative cystoscopy or indwelling ureteral catheter insertion. No critical difficulty was found in the intra-abdominal adhesions in patient 1. The simplicity and reproducibility of our procedure are its main advantages. We believe that this is a safe, feasible, effective, and minimally invasive approach, and therefore could be considered as the first-line treatment for RCU.

## Notes

New Insights & the Importance for the Pediatric Surgeon We present our laparoscopic technique for retrocaval ureter with a very minimally invasive procedure, demonstrating that it is an effective and safe treatment.
